# ZIC2 Is Essential for Maintenance of Latency and Is a Target of an Immediate Early Protein during Kaposi's Sarcoma-Associated Herpesvirus Lytic Reactivation

**DOI:** 10.1128/JVI.00980-17

**Published:** 2017-10-13

**Authors:** Yuanzhi Lyu, Kazushi Nakano, Ryan R. Davis, Clifford G. Tepper, Mel Campbell, Yoshihiro Izumiya

**Affiliations:** a Department of Dermatology, School of Medicine, University of California, Davis, Sacramento, California, USA; b Department of Biochemistry and Molecular Medicine, School of Medicine, University of California, Davis, Sacramento, California, USA; c University of California, Davis, Comprehensive Cancer Center, Sacramento, California, USA; University of Southern California

**Keywords:** Kaposi's sarcoma-associated herpesvirus, PRC2, ZIC2, epigenetic, reactivation, transcriptional regulation

## Abstract

Bivalent histone modifications are defined as repressive and activating epigenetic marks that simultaneously decorate the same genomic region. The H3K27me3 mark silences gene expression, while the H3K4me3 mark prevents the region from becoming permanently silenced and prepares the domain for activation when needed. Specific regions of Kaposi's sarcoma-associated herpesvirus (KSHV) latent episomes are poised to be activated by the KSHV replication and transcription activator (K-Rta). How KSHV episomes are prepared such that they maintain latent infection and switch to lytic replication by K-Rta remains unclear. K-Rta transactivation activity requires a protein degradation function; thus, we hypothesized that identification of cellular substrates of K-Rta may provide insight into the maintenance of KSHV latent infection and the switch to lytic replication. Here we show that a zinc finger protein, ZIC2, a key regulator for central nervous system development, is a substrate of K-Rta and is responsible for maintaining latency. K-Rta directly interacted with ZIC2 and functioned as an E3 ligase to ubiquitinate ZIC2. ZIC2 localized at immediate early and early gene cluster regions of the KSHV genome and contributed to tethering of polycomb repressive complex 2 through physical interaction, thus maintaining H3K27me3 marks at the K-Rta promoter. Accordingly, depletion of ZIC2 shifted the balance of bivalent histone modifications toward more active forms and induced KSHV reactivation in naturally infected cells. We suggest that ZIC2 turnover by K-Rta is a strategy employed by KSHV to favor the transition from latency to lytic replication.

**IMPORTANCE** Posttranslational histone modifications regulate the accessibility of transcriptional factors to DNA; thus, they have profound effects on gene expression (e.g., viral reactivation). KSHV episomes are known to possess bivalent chromatin domains. How such KSHV chromatin domains are maintained to be reactivatable by K-Rta remains unclear. We found that ZIC2, a transcriptional factor essential for stem cell pluripotency, plays a role in maintaining KSHV latent infection in naturally infected cells. We found that ZIC2 degradation by K-Rta shifts bivalent histone marks to a more active configuration, leading to KSHV reactivation. ZIC2 interacts with and maintains polycomb repressor complex 2 at the K-Rta promoter. Our findings uncover (i) a mechanism utilized by KSHV to maintain latent infection, (ii) a latency-lytic cycle switch operated by K-Rta, and (iii) a molecular mechanism of ZIC2-mediated local histone modification.

## INTRODUCTION

Kaposi's sarcoma-associated herpesvirus (KSHV), also designated human herpesvirus 8, has been linked to Kaposi's sarcoma (1–3). KSHV is also associated with two human lymphoproliferative diseases, primary effusion lymphoma (PEL) and AIDS-related multicentric Castleman's disease (4–7). Like all other herpesviruses, KSHV can exhibit two alternative life cycles, known as latency and lytic replication. KSHV reactivation is initiated by the expression of a single viral protein, the KSHV replication and transcription activator (K-Rta). K-Rta is both essential and sufficient to induce lytic reactivation of the latent KSHV genome in the BCBL-1 cell line model as well as in a *de novo* infection model (8–12). K-Rta is a potent transcription factor, with a putative N-terminal DNA-binding domain and a C-terminal transactivation domain (11, 13).

In addition to its function as a DNA-binding transcription factor, K-Rta is known to target cellular and viral proteins for protein degradation. The substrates include IRF7, K-RBP, Hey1, TRIF, HLA-DRα, and Myd88 (14–19). We have also shown that K-Rta recognizes a small ubiquitin-like modifier (SUMO) through SUMO-interacting motifs (SIM) and targets both SUMO peptides and SUMO-modified proteins for degradation (20). Mutation of the K-Rta SIM or the really interesting new gene (RING) finger-like domain significantly impairs its transactivation ability, linking the transactivation ability with the protein degradation function (20). These studies suggest that derepression through K-Rta-mediated protein degradation contributes to transactivation potency. This idea was first suggested by Yang et al. (15) in a study which demonstrated that K-Rta targets K-RBP, a zinc finger (ZnF) protein, for degradation and proposed that promotion of repressor degradation by viral transactivators may be a mechanism for lytic gene activation in the herpesvirus family (15).

ZnF proteins are among the most abundant proteins encoded by eukaryotic genomes. Nearly 3% of the human protein-coding sequence is estimated to encode ZnF proteins. ZnF domains are binding modules which recognize DNA, RNA, and protein. Accordingly, the functions of ZnF proteins are extraordinarily diverse, including roles in transcription repression/activation, RNA packaging, regulation of apoptosis, protein folding/assembly, and lipid binding (21, 22). In addition to K-RBP, other ZnF proteins are known to regulate KSHV gene expression. These proteins are KAP1 (TRIM28), YY1, PML (TRIM19), KZLP, and CTCF (23–30). Here, we identified ZIC2 (zinc finger protein of the cerebellum 2) as a key regulator of KSHV latency.

ZIC2 is one of five genes in the ZIC family. ZIC genes are involved in a variety of developmental processes, including neurogenesis, myogenesis, skeletal patterning, and left-right axis establishment. All five ZIC genes are highly conserved between mouse and human and share similar tandem repeats known as the C2H2 zinc finger motif (31). The loss of the DNA-binding ability of ZIC2 results in a loss of function (32, 33), suggesting that the DNA-binding activity of ZIC2 is essential for it to exert its function *in vivo*. Increased ZIC2 expression has also been documented in ovarian cancer cells (34) and pancreatic ductal adenocarcinoma (35). Moreover, ZIC2 is highly and specifically expressed in liver cancer stem cells (CSCs) and is essential for the self-renewal maintenance of liver CSCs (36). Similarly, ZIC2 is required for the proper differentiation of embryonic stem cells (ESCs) (37) by interacting with the nucleosome remodeling and histone deacetylation (NuRD) complex to regulate transcription of key developmental genes.

Similar to cellular chromosomes, latent KSHV episomes are organized into nucleosomes and viral gene expression is regulated by histone-modifying enzymes (38–40). Such histone modifications correlate with transcriptionally active, poised, and repressed domains of viral chromatin. Similar to promoters of key developmental genes in ESCs, KSHV immediate early (IE) and early (E) gene regions are decorated by bivalent histone modifications, which possess both H3K4me3 (an active mark) and H3K27me3 (a repressive mark) (39). By exhibiting both active and repressive features, bivalent genes are in a poised state, enabling them to be rapidly activated upon reactivation. Biochemically, the repressive H3K27me3 mark is deposited by polycomb repressive complex 2 (PRC2), which is composed of three core subunits, enhancer of zeste homolog 2 (EZH2), suppressor of zeste 12 homolog (SUZ12), and embryonic ectoderm development (EED). EZH2 trimethylates H3K27 via its histone methyltransferase activity (41). In mammals, the PRC2 complex is an important regulator involved in various biological processes, including cell proliferation, cancer development, and ESC pluripotency (42).

In this study, we identified ZIC2 to be a target of K-Rta for protein degradation. Genetic studies identified that ZIC2 binds to the K-Rta promoter region and regulates local histone modifications by tethering the PRC2 complex. Importantly, ZIC2 is necessary to maintain KSHV latency in PEL cells, as we found that ablation of ZIC2 expression alone is sufficient to trigger KSHV reactivation. We propose a model in which K-Rta targets ZIC2 to favor lytic gene expression.

## RESULTS

### Zinc finger transcription factor ZIC2 is a target of K-Rta for degradation.

**FIG 1:**
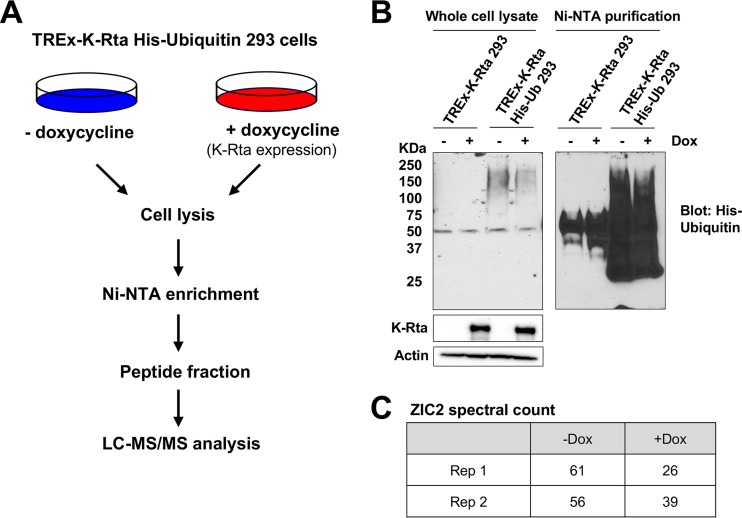
Identification of ZIC2 as a ubiquitinated target of K-Rta. (A) Schematic illustrating the identification of ubiquitinated target proteins of K-Rta using the TREx-K-Rta His-ubiquitin (Ub) 293 cell line. His-ubiquitin-conjugated proteins were enriched, followed by MS analysis. (B) Whole-cell lysates of TREx-K-Rta 293 cells and TREx-K-Rta His-ubiquitin 293 cells were subjected to nickel affinity chromatography and analyzed by immunoblotting with polyhistidine antibody. (C) Spectral count of ZIC2 protein identified in Ni-NTA pulldowns from untreated and Dox-treated TREx-K-Rta His-ubiquitin 293 cells. Rep, repeat assay.

K-Rta has been shown to degrade several cellular and viral proteins via an E3 ubiquitin ligase activity. To search for targets of K-Rta in an unbiased manner, we established a 293-derived cell line with inducible expression of FLAG- and hemagglutinin (HA) epitope-tagged K-Rta and stable expression of His-ubiquitin (TREx-K-Rta His-ubiquitin 293). We reasoned that substrates of K-Rta might show differences in the amount of ubiquitin conjugation upon K-Rta expression (Fig. 1A). We verified by immunoblotting that K-Rta expression was induced upon doxycycline (Dox) treatment (Fig. 1B, middle left). In addition, conjugation of His-tagged ubiquitin to cellular proteins in TREx-K-Rta His-ubiquitin 293 cells was also confirmed (Fig. 1B, top left). TREx-K-Rta 293 cells lacking exogenously expressed ubiquitin were used as a negative control.

To enrich His-ubiquitin-conjugated proteins, cell lysates were prepared from 10 150-mm dishes and ubiquitin-conjugated proteins were purified with Ni-nitrilotriacetic acid (NTA) agarose beads. Ubiquitination was confirmed by Western blotting using anti-His antibody (Fig. 1B, right). Proteins with molecular masses ranging from 25 to 200 kDa were detected in TREx-K-Rta His-ubiquitin 293 cells, indicating enrichment of His-ubiquitin-conjugated proteins (Fig. 1B, right). Ni-NTA pulldowns from TREx-K-Rta His-ubiquitin 293 cells were then subjected to liquid chromatography (LC)-tandem mass spectrometry (MS/MS). Proteins with a spectral count of >10 and a fold change in expression of >1.4 between samples not treated with Dox and samples treated with Dox are listed in Table S1 in the supplemental material. Among these proteins, the zinc finger transcription factor ZIC2 consistently showed a decrease in spectral counts upon Dox treatment in each of two independent experiments (Fig. 1C). ZIC2 is a major regulator of neuronal differentiation, and emerging evidence suggests that ZIC2 is required for ESC pluripotency (37, 43). Importantly, ZIC2 has been shown to regulate transcription through recruitment of histone-targeting enzymes at key developmental genes with bivalent histone marks (36, 37). Based on its function to maintain bivalent histone marks and demonstrated significance in cell development, we selected ZIC2 for further study to examine its role in the KSHV life cycle.

### K-Rta degrades ZIC2 through the ubiquitin-proteasome pathway.

**FIG 2:**
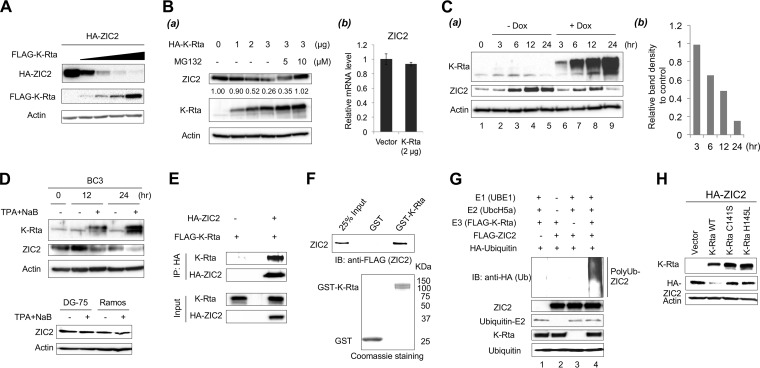
K-Rta interacts with ZIC2 and degrades ZIC2 in a proteasome-dependent manner. (A) 293T cells were cotransfected with a fixed amount of HA-tagged ZIC2 (1 μg) and increasing amounts of FLAG-K-Rta (0, 0.1, 0.2, 0.4, and 0.8 μg) for 24 h. Cell lysates were immunoblotted with anti-HA, anti-FLAG, or anti-β-actin antibody. (B) (a) 293T cells were transfected with increasing amounts of HA-K-Rta (0, 1, 2, and 3 μg). At 24 h posttransfection, cells were treated with dimethyl sulfoxide or MG132 (5 and 10 μM) for 12 h. The amount of endogenous ZIC2 expression was examined by immunoblotting. Values represent the signal intensity of the ZIC2 protein normalized to that of β-actin and compared to the signal obtained from control cells (0 μg of transfected HA-K-Rta). (b) 293T cells were transfected with 2 μg HA-K-Rta or the control vector, and the amount of ZIC2 mRNA was measured by RT-qPCR. (C) ZIC2 expression is decreased during KSHV lytic reactivation. TREx-F3H3-K-Rta BCBL-1 cells were induced with 1 μg/ml Dox for the indicated times. (a) Cell lysates were immunoblotted with anti-K-Rta, anti-ZIC2, or anti-β-actin antibody. (b) Quantification of ZIC2 protein levels shown in panel Ca. Values represent the intensity of the ZIC2 protein normalized to that of β-actin and compared to the signal obtained from the time-matched control cells (without Dox treatment). (D) (Top) BC3 cells were treated with 20 ng/ml TPA and 1 mM NaB for the indicated times. (Bottom) DG-75 and Ramos cells were also incubated with 20 ng/ml TPA and 1 mM NaB for 24 h. Cell lysates were prepared, and the indicated proteins were probed with specific antibodies. (E) 293T cells were cotransfected with FLAG-K-Rta and HA-tagged ZIC2 or the control vector. At 12 h posttransfection, the cells were treated with 10 μM MG132 for 24 h. Cell lysates were prepared and then immunoprecipitated with anti-HA antibody. Immunocomplexes and cell lysates were analyzed by Western blotting using anti-HA or anti-K-Rta antibody. (F) *In vitro* interaction of K-Rta and ZIC2 protein purified from Sf9 cells. Purified FLAG-ZIC2 was incubated with GST or GST-K-Rta immobilized on glutathione-Sepharose beads. ZIC2 protein bound to beads was analyzed by Western blotting using anti-FLAG antibody. (G) *In vitro* ubiquitination of ZIC2. E1, E2, HA-tagged ubiquitin, and FLAG-tagged K-Rta and ZIC2 proteins expressed in and purified from insect cells were used. The mixture containing the indicated components was incubated in reaction buffer at 37°C for 2.5 h. After quenching with SDS-PAGE loading buffer, the mixture was analyzed by Western blotting using specific antibodies. (H) 293 cells were cotransfected with HA-tagged ZIC2 and K-Rta or K-Rta mutants (C141S and H145L) for 24 h. Cell lysates were immunoblotted with anti-HA, anti-K-Rta, or anti-β-actin antibody. IB, immunoblotting; WT, wild type.

To confirm whether K-Rta is responsible for the degradation of ZIC2, a fixed amount of HA-ZIC2 was cotransfected with increasing amounts of FLAG-K-Rta, and ZIC2 protein levels were examined by immunoblotting. The results clearly showed that K-Rta decreased the amount of ectopic ZIC2 protein to nearly undetectable levels in a dose-dependent manner (Fig. 2A). Next, 293T cells were transfected with the K-Rta expression vector and the amount of endogenous ZIC2 was monitored. The results showed that the amount of endogenous ZIC2 was also decreased in the presence of K-Rta in a dose-dependent manner. Importantly, ZIC2 protein levels were recovered in the presence of the proteasome inhibitor MG132 (Fig. 2B). We noticed that there was a mobility shift of ZIC2 in the presence of MG132, indicating that ubiquitination might be a signal for the further posttranslational modification of ZIC2 (Fig. 2Ba). Reverse transcription-quantitative PCR (RT-qPCR) demonstrated that K-Rta expression did not affect ZIC2 mRNA levels significantly, indicating that the decreased levels of ZIC2 were due to posttranscriptional regulation (Fig. 2Bb). Next, we examined ZIC2 degradation in the context of KSHV reactivation. Two different PEL cell lines were used in order to avoid the potential bias introduced by the methods utilized to trigger the KSHV reactivation or cell line variation. Reactivation of KSHV from TREx-F3H3-K-Rta BCBL-1 cells was induced with 1 μg/ml Dox (Fig. 2Ca). Interestingly, we found that the ZIC2 protein levels in untreated control TREx-F3H3-K-Rta BCBL-1 cells were changed during culture. ZIC2 protein levels rose and peaked at 12 h after the cells were plated into fresh culture medium and decreased slightly at 24 h. This suggests that the expression of ZIC2 protein is sensitive to the tissue culture environment (Fig. 2Ca). ZIC2 protein levels were reduced as early as 6 h in K-Rta-expressing cells compared to the levels in the time-matched control cells (Fig. 2Cb). At 24 h after Dox treatment, the amount of ZIC2 protein was decreased to nearly undetectable levels, while it was still maintained at a high level in control cells (Fig. 2Cb). BC3 cells were also used, and reactivation was triggered by treatment with a combination of 20 ng/ml tetradecanoyl phorbol acetate (TPA) and 1 mM sodium butyrate (NaB). Consistent with the findings for K-Rta-inducible cells, ZIC2 protein levels were reduced by KSHV reactivation in BC3 cells compared with the levels in untreated cells (Fig. 2D, top). ZIC2 protein levels were not changed at 24 h posttreatment with TPA-NaB in either KSHV-negative DG-75 or Ramos cells (Fig. 2D, bottom).

Next, we examined the physical interaction between K-Rta and ZIC2. 293T cells were cotransfected with FLAG-K-Rta and HA-ZIC2, and the transfected cells were cultured in the presence of MG132. The cell lysates were harvested and immunoprecipitated with anti-HA antibody. The results showed that K-Rta was coimmunoprecipitated with HA-ZIC2 (Fig. 2E). We further examined the direct interaction between ZIC2 and K-Rta. FLAG-tagged ZIC2 protein purified from baculovirus-infected Sf9 insect cells was incubated with glutathione *S*-transferase (GST) or GST-K-Rta immobilized on glutathione-Sepharose beads. The results showed that ZIC2 directly interacted with GST-K-Rta but not with GST (Fig. 2F). Since K-Rta possesses ubiquitin E3 ligase activity, we next examined if K-Rta acts as an E3 ligase of ZIC2. FLAG-tagged K-Rta protein was expressed and purified from Sf9 cells. *In vitro* ubiquitination reactions showed that K-Rta catalyzed the polyubiquitination of ZIC2 only in the presence of the E1 and E2 enzymes (Fig. 2G). Finally, it has been reported that the Cys/His-rich domain (amino acids 118 to 207) of K-Rta is important for targeting IRF7 degradation (14). We cotransfected HA-ZIC2 and K-Rta mutants (C141S and H145L) in 293 cells and found that both mutants largely lost the ability to degrade ZIC2 (Fig. 2H). Taken together, these results suggested that K-Rta functions as a ubiquitin E3 ligase of ZIC2 and downmodulates ZIC2 in a proteasome-dependent manner.

### ZIC2 suppresses KSHV lytic replication in iSLK.219 cells.

**FIG 3:**
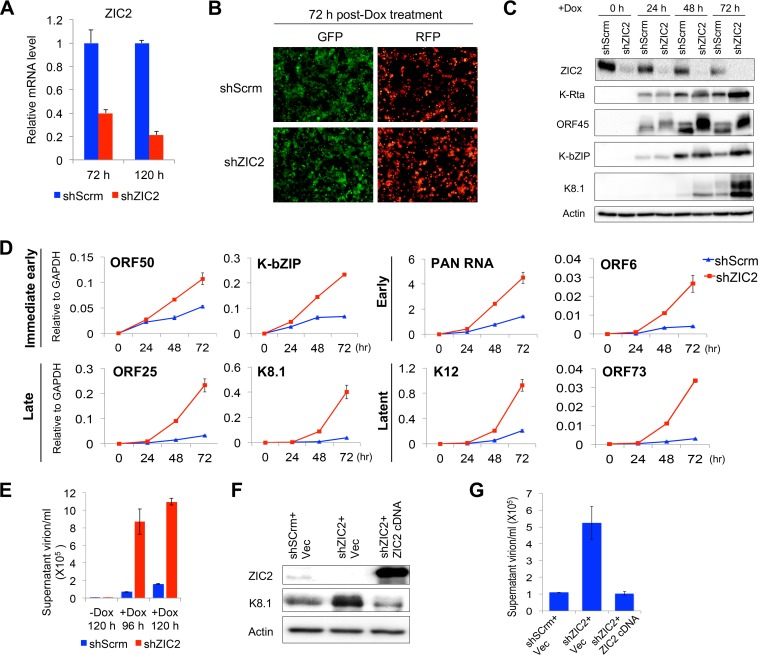
ZIC2 represses KSHV lytic reactivation in iSLK.219 cells. (A) iSLK.219 cells were transduced with lentivirus expressing shScrm or shZIC2. ZIC2 mRNA levels were measured by RT-qPCR at 72 and 120 h postransduction. (B to E) At 3 days postransduction, cells were treated with 1 μg/ml Dox for various time periods. (B) GFP and RFP were monitored at 72 h after Dox treatment. (C) At 0, 24, 48, and 72 h after Dox treatment, the cells were collected and whole-cell proteins were extracted. The levels of the ZIC2, K-Rta, ORF45, K-bZIP, and K8.1 proteins were examined by immunoblotting. (D) Total RNA was harvested at the indicated time points, and viral gene expression was measured by RT-qPCR. The viral gene names are shown, and GAPDH was used as an internal control. (E) iSLK.219 cells were treated as described in the legend to panel B. At 96 and 120 h after Dox treatment, the medium was collected, virion DNAs were extracted, and the copy number of virion DNA was detected by RT-qPCR. (F and G) iSLK.219 cells were simultaneously transduced with lentivirus expressing shScrm or shZIC2 and control lentivirus or lentivirus expressing shRNA-resistant ZIC2 cDNA. At 3 days postransduction, cells were treated with 1 μg/ml Dox for 96 h. (F) Cell lysates were immunoblotted with anti-ZIC2, anti-K8.1, or anti-β-actin antibody. (G) The medium was collected, virion DNAs were extracted, and the copy number of virion DNA was quantified by RT-qPCR. Vec, vector.

Our biochemical studies demonstrated that K-Rta interacts with and degrades ZIC2. Why does K-Rta want to target ZIC2 for degradation? What is the function of ZIC2 in the KSHV life cycle? To answer these questions, the ZIC2 in iSLK.219 cells was specifically knocked down with short hairpin RNA (shRNA). RT-qPCR analyses confirmed that ZIC2 mRNA was depleted more than 60% and nearly 80% at 72 h and 120 h postransduction with lentivirus expressing shRNA specific for ZIC2 (shZIC2) (Fig. 3A). After 72 h of shZIC2 transduction, cells were treated with 1 μg/ml Dox and viral gene expression and replication were monitored by a series of experiments. KSHV reactivation was first monitored by expression of red fluorescent protein (RFP), which is an indicator of PAN RNA (lytic) promoter activation (Fig. 3B). The results showed brighter RFP signals in ZIC2-knockdown (KD) cells at 72 h after Dox treatment, although the number of RFP-positive cells was not significantly different from the number of reactivated control cells (Fig. 3B). Second, lytic viral protein and mRNA expression was examined by immunoblotting (Fig. 3C) and RT-qPCR (Fig. 3D) during the course of reactivation. Consistent with the increase in the RFP signal intensity, the expression of lytic proteins K-Rta, ORF45 (an IE gene), K-bZIP (an E gene), and K8.1 (a late gene) was increased in ZIC2-KD cells at 48 and 72 h after Dox treatment (Fig. 3C). We noticed a mobility shift of ORF45 in ZIC2-KD cells (Fig. 3C), which was likely due to phosphorylation (44). The transcriptional levels of KSHV genes of all four kinetic classes were also examined (Fig. 3D). ZIC2 KD enhanced IE and E viral gene expression by 2- to 5-fold and enhanced late gene expression by approximately 10-fold at 72 h postinduction compared with the levels of gene expression for the controls (i.e., scrambled shRNA [shScrm]-transduced cells). The latent genes K12 and ORF73 were also upregulated by about 5- and 11-fold, respectively (Fig. 3D). Increased viral gene expression was accompanied by enhanced virion production in supernatants at later time points, which demonstrated a 12-fold increase in the number of virus particles in ZIC2-KD cells at 96 h postinduction (Fig. 3E). To eliminate possible off-target effects of the shRNA, we generated a lentivirus vector expressing an shRNA-resistant ZIC2 cDNA and performed a complementation experiment by introducing the cDNA into ZIC2-KD cells. The effect of ZIC2 KD on KSHV lytic replication, monitored by measurement of lytic protein levels and virion production, was strongly reversed by exogenous ZIC2 expression (Fig. 3F and G). Taken together, these results demonstrate that ZIC2 acts as a repressor of KSHV lytic gene expression during reactivation.

### ZIC2 maintains KSHV latency in PEL cells.

**FIG 4:**
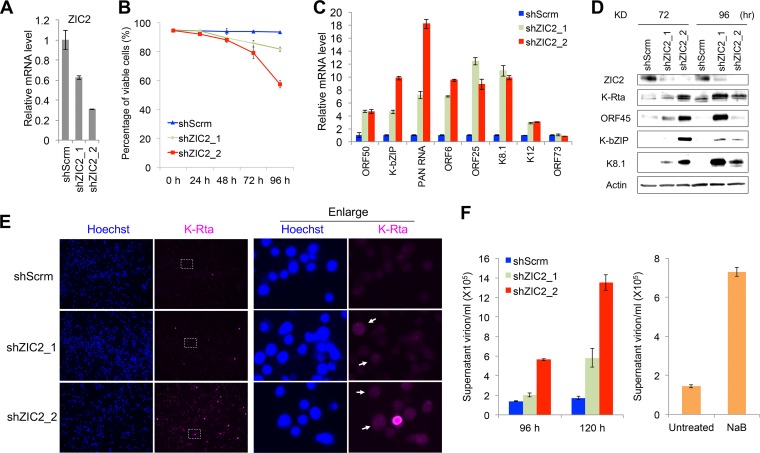
ZIC2 is essential for maintaining KSHV latent infection in BCBL-1 cells. (A) BCBL-1 cells were transduced with lentivirus expressing shScrm, shZIC2_1, or shZIC2_2. Total RNA was harvested at 72 h postransduction, and ZIC2 expression was measured by RT-qPCR. (B) At 0, 24, 48, 72, and 96 h postransduction, cell viability was measured by a trypan blue exclusion assay. (C) Viral gene expression was measured by RT-qPCR at 96 h postransduction. The relative levels of mRNA were normalized to the level of GAPDH. (D) BCBL-1 cells were transduced for 72 and 96 h. Cell lysates were immunoblotted with anti-ZIC2, anti-K-Rta, anti-ORF45, anti-K-bZIP, anti-K8.1, or anti-β-actin antibody. (E) IFA with anti-K-Rta antibody (far-red fluorescence) was performed at 72 h after lentiviral transduction to detect lytic reactivation. White rectangles, the areas shown in the enlarged views on the right; white arrowheads, ZIC2-KD cells with mild induction of K-Rta expression. (F) (Left) BCBL-1 cells were transduced for 96 and 120 h; (right) BCBL-1 cells were treated with 1 mM NaB for 120 h. The culture supernatant was collected, virion DNAs were extracted, and the copy number of virion DNA was quantified by RT-qPCR.

Next, we studied the roles of ZIC2 on KSHV reactivation in naturally infected cells. Initially, we tried to stably knock down ZIC2 in PEL cells; however, we could not obtain a cell population that showed reduced ZIC2 expression. Subsequent studies found that ZIC2 KD alone induced lytic reactivation in BCBL-1 cells and eventually killed the ZIC2-KD cells. The results were confirmed with two different ZIC2-specific shRNAs (shZIC2_1 and shZIC2_2) (Fig. 4A and B). The results showed that knockdown of ZIC2 by both shRNAs induced the expression of lytic genes (Fig. 4C). Immunoblotting for K-Rta, ORF45, K-bZIP, and K8.1 further confirmed the production of lytic proteins after ZIC2 KD (Fig. 4D). We further examined the expression of K-Rta by immunofluorescence assay (IFA) at 72 h after ZIC2 KD (Fig. 4E). Compared to the number of K-Rta-positive control cells, an increased number of K-Rta-positive ZIC2-KD cells (far-red fluorescence) was observed (Fig. 4E, left). We also observed that almost all of the shZIC2_2-transduced cells showed low but noticeable signals of K-Rta compared with the levels in shScrm-transduced cells (Fig. 4E, enlarged view), indicating that ZIC2 is required for the suppression of K-Rta expression. As expected, the level of KSHV virion production in the supernatant was elevated in the ZIC2-KD cells. We used NaB-treated BCBL-1 cells as a positive control (45, 46). Induction with NaB resulted in a 5-fold increase in the level of virion production at 120 h posttreatment compared to that in untreated BCBL-1 cells (Fig. 4F, right). ZIC2 KD resulted in 4- and 8-fold increases in the levels of virion production by shZIC2_1- and shZIC2_2-transduced cells, respectively, compared to that in shScrm-transduced cells at 120 h postransduction (Fig. 4F, left). Consistent with the higher knockdown efficiency of shZIC2_2, we found that shZIC2_2 induced lytic proteins earlier and resulted in the production of more virus particles at both 96 and 120 h compared with the results for shZIC2_1 (Fig. 4A).

### Genome-wide mapping of ZIC2-binding sites on KSHV episomes.

**FIG 5:**
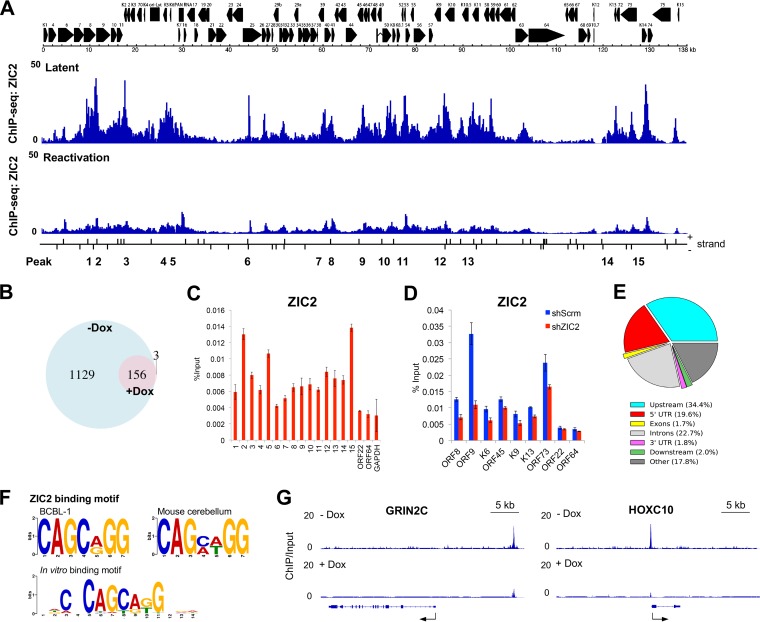
Genome-wide distribution of ZIC2 on the latent KSHV genome. (A) ChIP-Seq analysis of ZIC2 binding to the latent KSHV genome and Dox-induced (1 μg/ml, 24 h) TREx-F3H3-K-Rta BCBL-1 cells. ChIP enrichment signals were normalized to those derived from the input DNA control. The positions of the predicted ZIC2 recognition motif [CAGC(A/G)GG] on the KSHV genome are also indicated. Short vertical lines denote the corresponding positions of the ZIC2 motif on the two DNA strands of the KSHV genome. (B) Venn diagram illustrating the overlap between ZIC2 peaks on the host genome from ChIP-Seq with latent and lytic reactivated BCBL-1 cells. (C) Verification of the ChIP-Seq result by ChIP-qPCR analysis of the indicated genes/regions in BCBL-1 cells. (D) Verification of the ChIP-Seq result by ChIP-qPCR analysis in ZIC2-KD cells. iSLK.219 cells were transduced with lentivirus expressing shScrm or shZIC2. ChIP-qPCR assays were performed to determine the level of ZIC2 binding on the indicated regions at 72 h postransduction. (E) Genome-wide distribution of ZIC2 in latent BCBL-1 cells. Genomic distribution of ZIC2 enrichment peaks throughout the human genome determined by ChIP-Seq analysis. The upstream and downstream lengths for annotation were set to 3 kb flanking the gene transcript start and termination sites. UTR, untranslated region. (F) Identification of a ZIC2 recognition motif. ZIC2-binding motifs were analyzed from ZIC2 ChIP-Seq data from latent BCBL-1 cells and data from a developing mouse cerebellum (GEO accession number GSM1486431) using the MEME suite of programs. The E value was <10^−10^. The *in vitro* mouse Zic2 protein binding motif from the JASPAR database (accession number PB0206.1) is also shown. (G) Genome browser tracks showing ZIC2 at the *GRIN2C* and *HOXC10* genomic loci in latent and lytic reactivated BCBL-1 cells.

To understand the molecular action of ZIC2, we next determined ZIC2-binding sites on the KSHV genome with chromatin immunoprecipitation-sequencing (ChIP-Seq) analysis in BCBL-1 cells. Latent and lytic reactivated TREx-F3H3-K-Rta BCBL-1 cell chromatin DNA was enriched by immunoprecipitation with anti-ZIC2 antibodies, followed by library preparation and sequencing on an Illumina platform. Approximately 50 million sequencing reads were obtained. Among those sequencing reads, 97.6% and 97.0% of the tags derived from the latent and lytic reactivated cells were aligned to the human genome reference (hg19), respectively, and 1.1% of sequences were aligned to the KSHV genome (NCBI GenBank accession number GQ994935) (Fig. 5A). Peaks representing loci with enriched binding of ZIC2 were identified using model-based analysis of the ChIP-Seq data with the MACS2 program with a false discovery rate (FDR; *q* value) cutoff value of 0.05, and the results were normalized to those for the input DNA control. On the latent KSHV genome, the results showed that ZIC2 preferentially binds to IE and E gene cluster regions (1 to 30 kb and 60 to 95 kb, respectively) and the latent gene region (120 to 130 kb), while ZIC2 peaks were largely depleted from late gene clusters, such as ORF22- and ORF64-coding regions (Fig. 5A). ZIC2-binding peaks were significantly reduced in lytic reactivated BCBL-1 cells (Fig. 5A). Similarly, the number of ZIC2-specific binding peaks on the host genome were decreased sharply from 1,285 to 159 by induction of KSHV reactivation (Fig. 5B). Importantly, 156 of the peaks identified in reactivated BCBL-1 cells (98.1%) were among the peaks found in latent BCBL-1 cells (Fig. 5B).

Based on the ChIP-Seq results for latent BCBL-1 cells, we designed 15 specific primer sets corresponding to the peaks shown in Fig. 5A and performed ChIP-qPCR analyses. The cellular GAPDH (glyceraldehyde-3-phosphate dehydrogenase) promoter was included as a negative control. Chromatin immunoprecipitation (ChIP)-quantitative PCR (qPCR) confirmed that ZIC2 was enriched on all the peak-containing regions identified in latent BCBL-1 cells (Fig. 5C). A similar ZIC2-binding profile was obtained for iSLK.219 cells by ChIP-qPCR analyses, and knockdown of ZIC2 demonstrated lower levels of ZIC2 binding at these sites, confirming that the observed peaks are indeed the result of ZIC2 binding (Fig. 5D).

With peak annotation and visualization (PAVIS), more than one-third of the peaks on the host genome were located within 3 kb upstream of the transcription start site (TSS) (Fig. 5E). To further validate our ChIP-Seq data, a consensus ZIC2-binding motif was studied. The human genomic sequences flanking ±50 bp from the center of all the ZIC2 peaks were retrieved and analyzed by use of the MEME suite of programs and compared with publicly available Zic1/2 ChIP-Seq data from a developing mouse cerebellum (GEO accession number GSM1486431). Identified motifs are presented in a Logo format (Fig. 5F). We found that ZIC2 binds to the CAGC(A/G)GG sequence motif in BCBL-1 cells (E value, <10^−10^), which is highly similar to the findings for the motif identified in the mouse cerebellum (Fig. 5F). Remarkably, the ZIC2-binding motifs based on the results of our ChIP-Seq analysis coincided with the central sequence of the *in vitro* Zic2-binding motif (JASPAR database accession number PB0206.1) (Fig. 5F). Taken together, these results validated the authenticity and location of the ZIC2-binding sites on the KSHV genome. With respect to specific cellular genes, our ChIP-Seq results also showed that ZIC2 binding is associated with regions surrounding the *GRIN2C* and *HOXC10* genes (Fig. 5G), both of which are essential for neural development, which is consistent with findings presented in previous reports (37, 47).

### ZIC2 depletion alters histone modifications at the promoter of K-Rta.

**FIG 6:**
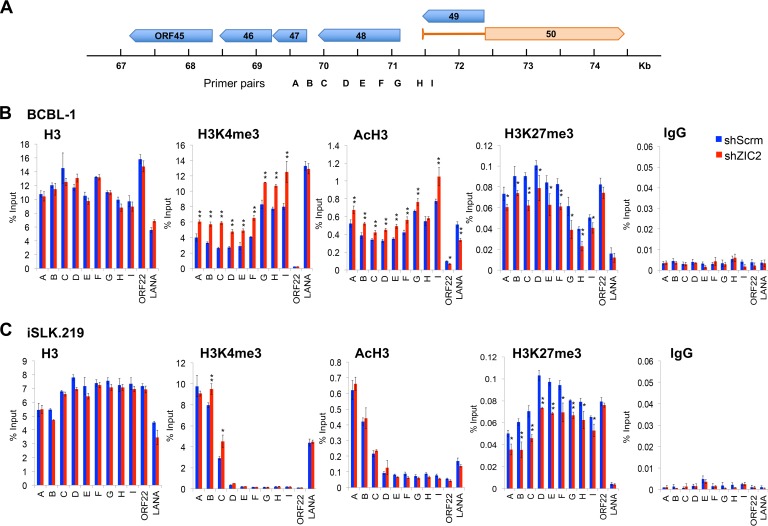
ZIC2 maintains H3K27me3 marks at the genomic region harboring bivalent histone marks. (A) Positions of qPCR primer pairs (specific for regions A to I) on the K-Rta promoter used for ChIP assays. (B) BCBL-1 cells were infected with lentivirus expressing shScrm or shZIC2 for 72 h. ChIP-qPCR analyses of the selected regions were performed using antibodies against H3, H3K4me3, AcH3, and H3K27me3. (C) iSLK.219 cells were infected with lentivirus expressing shScrm or shZIC2 for 72 h. The localization of H3, H3K4me3, AcH3, and H3K27me3 on the K-Rta promoter was detected by ChIP-qPCR assays. Normal rabbit IgG was used as a negative control. The data are presented as means ± SDs. *, *P* < 0.05; **, *P* < 0.01.

With the knowledge of the locations of the ZIC2 recruitment sites on the KSHV genome, we used the K-Rta promoter region as a model to study the relationship between ZIC2 binding and local histone modifications in BCBL-1 cells. K-Rta promoter activity is critical for KSHV reactivation; thus, we focused on histone modification on the K-Rta promoter region. Antibodies that recognize specific histone modifications (H3K4me3, AcH3, and H3K27me3) were used in ChIP-qPCR assays to monitor the changes induced by ZIC2 KD. Nine primer pairs (primer pairs specific for regions A to I) were designed to cover the K-Rta promoter (regions A to G, ∼1,900 to 300 bp upstream from the TSS), the TSS (region H), and the intron region (region I) (Fig. 6A). We also included the ORF22-coding region and the LANA promoter region as controls in the ChIP-qPCR assays, as these regions have been shown to display different histone marks (39). BCBL-1 cells were transduced with shScrm or shZIC2 for 3 days. Consistent with the findings of previous studies (39, 48), the K-Rta promoter region was occupied by both active H3K4me3 and active AcH3 histone marks and the repressive H3K27me3 histone mark in control BCBL-1 cells (Fig. 6B, blue). In contrast, the ORF22-coding region was occupied only by the repressive H3K27me3 histone mark, while the LANA promoter region was occupied only by active H3K4me3 and AcH3 histone marks (Fig. 6B). We found that ZIC2 KD (Fig. 6B, red) increased the levels of both the H3K4me3 and AcH3 marks and reduced the level of the H3K27me3 mark from K-Rta promoter regions, while the total histone H3 occupancy on these regions was not significantly affected by ZIC2 KD. These results indicate that ZIC2 maintains KSHV latency by preserving a relatively low level of active H3K4me3 and AcH3 marks and high level of the repressive H3K27me3 mark on the K-Rta promoter.

### ZIC2 is required for PRC2-mediated deposition of the H3K27me3 mark on the K-Rta promoter.

The depletion of ZIC2 resulted in induction of lytic replication in BCBL-1 cells (Fig. 4), which makes it difficult to assess the direct effects of ZIC2 KD. On the other hand, ZIC2 KD alone did not reactivate KSHV lytic replication in iSLK.219 cells (Fig. 3C and D, 0 h); thus, we next used iSLK.219 cells to examine the direct effects of ZIC2 KD on local histone modifications. iSLK.219 cells were transduced with shScrm or shZIC2 for 3 days, followed by ChIP-qPCR analyses for H3K4me3, AcH3, and H3K27me3. In contrast to the findings for BCBL-1 cells, we found that the K-Rta promoter region (regions D to I, spanning from ∼1,200 bp upstream of the TSS to the intron region) showed little H3K4me3 and AcH3 histone modifications in iSLK.219 cells (Fig. 6C, blue). ZIC2 KD had no effect on H3K4me3 and AcH3 marks in regions D to I (Fig. 6C). However, similar to BCBL-1 cells, we found a significant decrease in the amount of the H3K27me3 mark on the K-Rta promoter by ZIC2 KD (Fig. 6C). Given the differences in histone modification at K-Rta promoter regions between BCBL-1 and iSLK.219 cells, the increase in the amounts of the H3K4me3 and AcH3 marks in ZIC2-KD BCBL-1 cells is likely due to reactivation initiation but is not the direct consequence of ZIC2 KD (Fig. 6B). Interestingly, the far-upstream promoter of K-Rta (regions B and C, ∼1,800 to 1,400 bp upstream of TSS) had a relatively high level of the H3K4me3 mark in iSLK.219 cells, and the modification was increased by ZIC2 KD (Fig. 6C).

**FIG 7:**
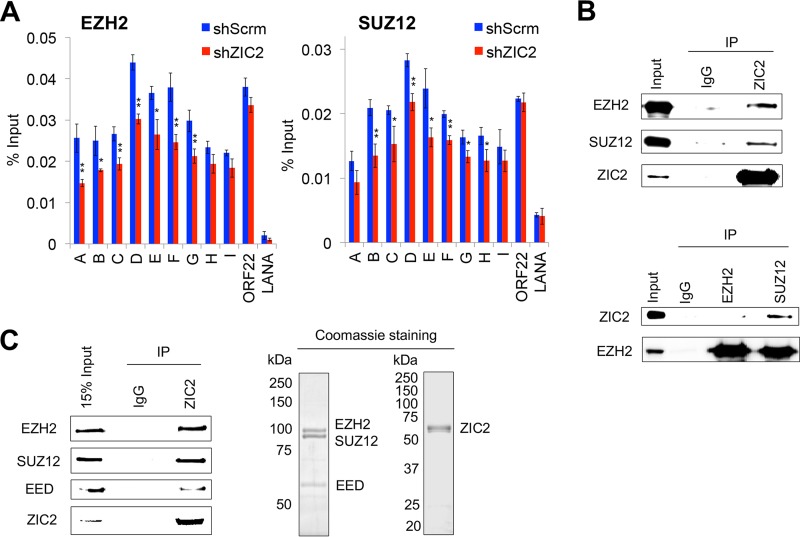
ZIC2 is required for the PRC2-mediated deposition of the H3K27me3 mark on the K-Rta promoter. (A) iSLK.219 cells were treated as described in the legend to Fig. 6C. ChIP-qPCR analyses of the selected regions were performed using antibodies against EZH2 and SUZ12. The data are presented as means ± SDs. *, *P* < 0.05; **, *P* < 0.01. (B) Endogenous immunoprecipitation (IP) followed by Western blotting shows the interaction of ZIC2 with EZH2 and SUZ12 in BCBL-1 cells. (Top) ZIC2 IP followed by EZH2 and SUZ12 Western blotting; (bottom) EZH2 and SUZ12 IP followed by ZIC2 Western blotting. (C) *In vitro* interaction of ZIC2 with the PRC2 complex (EZH2, SUZ12, and EED) purified from Sf9 cells. (Left) Purified FLAG-ZIC2 was incubated with the PRC2 complex (FLAG-EZH2, FLAG-SUZ12, and FLAG-EED). The interaction was probed with control IgG or anti-ZIC2 antibody, precipitated proteins were analyzed by Western blotting using antibodies against EZH2, SUZ12, and EED. (Right) Purified ZIC2 and PRC2 proteins were subjected to SDS-PAGE and stained with Coomassie brilliant blue.

As the PRC2 complex is responsible for the deposition of the repressive H3K27me3 mark, we further examined the localization of EZH2 and SUZ12 on the K-Rta promoter by a ChIP assay. EZH2 and SUZ12 colocalized with the H3K27me3 mark in shScrm-transduced iSLK.219 cells, with the strongest signals being found at regions D to F of the K-Rta promoter (∼1,200 to 600 bp upstream of the TSS) (Fig. 7A, blue). The binding of EZH2 to the K-Rta promoter (regions A to G, ∼1,900 to 300 bp upstream of the TSS) was inhibited in ZIC2-KD cells (Fig. 7A, left). Similarly, the binding of SUZ12 to the K-Rta promoter (regions B to H, ∼1,800 bp upstream of the TSS to the TSS) was also decreased in ZIC2-KD cells (Fig. 7A, right). These results correspond well with the diminished levels of the H3K27me3 marks present at these regions (Fig. 6).

Knowing that ZIC2 is required for the localization of PRC2/H3K27me3 on the K-Rta promoter, we speculated that ZIC2 interacts with PRC2 proteins and contributes to tethering of the PRC2 complex at the genomic sites. Accordingly, interactions between ZIC2 and PRC2 were examined by coimmunoprecipitation of the endogenous proteins in BCBL-1 cells. The results showed that a ZIC2 antibody but not control IgG precipitated both EZH2 and SUZ12 (Fig. 7B, top). Reciprocal experiments further verified the interaction of ZIC2 with the PRC2 complex (Fig. 7B, bottom). Finally, the interaction between the ZIC2 and PRC2 proteins was examined *in vitro* with purified proteins; this eliminates the possibility that the interaction is mediated by chromatin. All ZIC2 and PRC2 complex proteins (EZH2, SUZ12, and EED) were purified from baculovirus-infected Sf9 insect cells (Fig. 7C, right). The results showed a direct interaction between the ZIC2 and PRC2 proteins (Fig. 7C, left). Taken together, these results indicate that ZIC2 maintains the localization of the PRC2 complex through physical interaction and is required for PRC2-mediated deposition of H3K27me3 on the K-Rta promoter and, therefore, maintenance of latent infection.

## DISCUSSION

**FIG 8:**
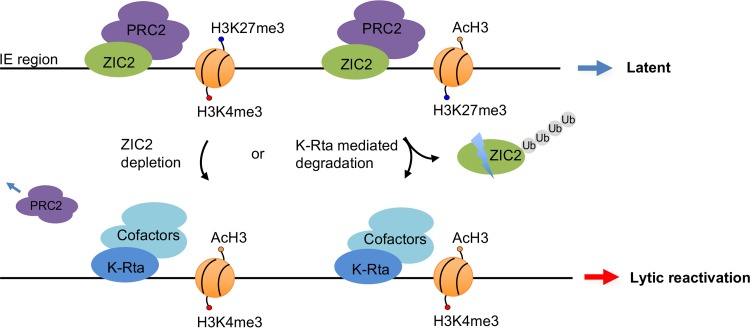
Proposed model of a regulatory loop between ZIC2-mediated repression and K-Rta-dependent activation of KSHV lytic replication. In latent KSHV-infected cells, ZIC2 interacts with the PRC2 complex and maintains the localization of PRC2 on the immediate early (IE) gene region, setting a poised state of IE genes. Depletion of ZIC2 and/or K-Rta-mediated ZIC2 degradation leads to the derepression of IE genes by decreasing the amount of H3K27me3 marks at the genomic sites, which results in KSHV lytic reactivation. K-Rta expression further promotes ZIC2 for degradation and recruitment of transcriptional cofactors to maximize lytic gene expression.

Previous studies showed that transcriptional activation is frequently associated with the protein degradation function and that the transactivator region often possesses a degron, a domain responsible for protein degradation. This was first examined in detail for the Saccharomyces cerevisiae GCN4 transcriptional activator (49, 50). Transactivators possess such a domain either in their own protein sequence or through physical interaction with proteasome-associated proteins (49). The K-Rta transactivation function associates with a protein degradation function, and inhibition of this activity significantly diminishes transactivation ability (20). This is likely due to degradation of specific repressors on the genomes, facilitation of RNA polymerase II recycling, and/or promoter clearance (49). In this study, we identified ZIC2 to be one of the targets of K-Rta. Importantly, we demonstrated that ZIC2 is essential for maintaining KSHV latency in naturally infected cells. ZIC2 is required for the PRC2-mediated deposition of repressive histone marks on IE gene regions of the KSHV genome, and ablation of ZIC2 by shRNA- or K-Rta-mediated degradation increased the level of KSHV gene expression (Fig. 8).

Similar to K-Rta, herpes simplex virus 1 ICP0, a viral ubiquitin E3 ligase, also targets multiple zinc finger proteins (51). Of note, RING finger motifs are atypical ZnF domains that are frequently conserved in E3 ubiquitin ligases. In this study, besides ZIC2, several other ZnF proteins, YY1, ZIP6, and GPATCH8, showed differences in the levels of ubiquitination in the presence of K-Rta (see Table S1 in the supplemental material). Considering that a large fraction of cellular open reading frames (ORFs) encodes ZnF transcription factors, these ZnF proteins may function as scaffolds by interacting with chromatin remodelers and specific DNA sequences to coordinate gene expression programs in a tissue and cellular environment-specific manner. Thus far, several ZnF proteins, including K-RBP, KAP1 (TRIM28), YY1, PML (TRIM19), and CTCF, have been shown to regulate KSHV gene expression (23–30, 52), and these molecules are known to participate in epigenetic gene regulation (52–54). Similar to ZIC2, silencing of CTCF or K-RBP also enhances K-RTA-mediated KSHV lytic replication (24, 25).

Our large-scale isolation of ubiquitinated proteins was performed in the absence of proteasome inhibitors (e.g., MG132), after consideration of its global effects, including K-Rta expression itself. Accordingly, we are likely missing a large fraction of less abundant proteins that were degraded within 24 h of K-Rta induction before sample preparation. Detailed proteomics studies with a number of controls and mutant K-Rta proteins would provide a more complete list. With our approach, we found that the amount of isolated His-ubiquitin-conjugated proteins was lower in the K-Rta-expressing cells than in control cells (Fig. 1B), indicating that ZIC2 is naturally ubiquitinylated in the absence of K-Rta and K-Rta increased ZIC2 turnover before cell lysates were harvested (24 h).

In the course of our experiments, we found that histone modifications on the K-Rta promoter region are different between BCBL-1 and iSLK.219 cells (Fig. 6B and C). The difference correlates well with the outcome of KSHV reactivation (Fig. 3 and 4). We found that ZIC2 KD can reactivate KSHV in BCBL-1 cells, where the levels of the H3K4me3 and AcH3 marks on the K-Rta promoter are significantly higher than those in iSLK.219 cells (Fig. 6B and C). Because the K-Rta promoter is silent but poised to be activated, reduction of the repressive H3K27me3 mark on the K-Rta promoter alone could trigger lytic reactivation in BCBL-1 cells (Fig. 4 and 6B). However, in iSLK.219 cells, the K-Rta promoter (regions D to I) was strongly repressed and lacked bivalency, in that active histone marks were absent; thus, decreasing the amount of the H3K27me3 mark by ZIC2 KD alone could not shift to the virus lytic gene expression (Fig. 3C and D, 0 h, and 6C). As our ChIP analyses were performed with a population of cultured cells, the results indicated that the majority of iSLK.219 cells in culture were not readily reactivatable. Accordingly, additional stimuli, such as histone deacetylase inhibitors (e.g., sodium butyrate), TPA, and/or exogenous K-Rta for recruitment of histone acetylases (55, 56), are necessary for endogenous K-Rta expression in iSLK.219 cells.

Our ChIP-Seq study identified ZIC2-binding sites on both cellular and KSHV chromosomes. We noticed that the ZIC2 recognition motif identified on the host genome in BCBL-1 cells is similar to that in the mouse cerebellum, as well as to the *in vitro* Zic2-binding motif (Fig. 5F). The KSHV genome contains a total of 54 putative ZIC2-binding sites [CAGC(A/G)GG] (Fig. 5A). Several positions (e.g., ORF8, ORF9, ORF45, and K8) containing the consensus motif were indeed bound by ZIC2. However, the consensus motif was not found in the ZIC2-binding LANA promoter region (Fig. 5A), suggesting that ZIC2 might also be recruited to the genome through indirect mechanisms. We noticed that the late gene regions also contain the ZIC2-binding motif without ZIC2 recruitment, which is partly due to having a strong heterochromatin structure at the region during latency (39, 48, 57) (Fig. 5A). In addition, we noticed ZIC2 localization at the LANA promoter region without the significant recruitment of PRC2. The absence of the PRC2 complex on the LANA promoter corresponds well with the lower H3K27me3 signal at this region (Fig. 6 and 7), and ZIC2 KD reduced the AcH3 mark on the LANA promoter in BCBL-1 cells (Fig. 6B). The results indicate that ZIC2 does not constitutively interact with the PRC2 complex. As suggested by others, ZIC2-interacting partners may vary depending on the local chromatin environment. It has been shown that ZIC2 acts as an important activator, recruiting the nuclear remodeling factor (NURF) complex to initiate Oct4 expression and maintain self-renewal in liver cancer stem cells (36). ZIC2 also binds to open DNase I-hypersensitive sites to facilitate gene expression in the developing cerebellum (47). Transcriptome sequencing analyses also demonstrated that similar numbers of genes are up- and downregulated in ZIC2-KD ESCs (37). An interaction between the NuRD complex and ZIC2 has also been demonstrated, and the association is important for the maintenance of H3K27me3 on the Hoxc cluster (37).

Toth et al. found that the viral latent protein LANA can interact with and recruit PRC2 to the KSHV genome during *de novo* infection (58). We have now identified a cellular regulator which participates in the maintenance of bivalent chromatin marks and gene regulation in the IE and E regions. Our results show that ZIC2 maintains the localization of PRC2 in the KSHV IE gene region in latently infected cells, which expands our knowledge of how PRC2/H3K27me3 is deposited to this gene cluster. Similar mechanisms were reported in mouse ESCs. Zic2 sets a poised state on developmental genes, and the knockdown of Zic2 broadly reduces the H3K27me3 mark on the Hoxc gene cluster (37). In our study, we further demonstrated that ZIC2 could interact with the PRC2 complex to maintain H3K27me3 marks, providing a molecular mechanism. ZIC2-binding sites significantly overlapped LANA-binding sites on the latent KSHV genome (59). It is interesting to speculate that viral latent proteins and ZIC2 may function together to inhibit KSHV lytic reactivation.

Induction of cell differentiation frequently induces herpesvirus reactivation; however, how cell differentiation triggers reactivation varies among herpesviruses and the cell lines used. For example, the transcription factor KLF4, which is known to promote epithelial cell differentiation, induces differentiation-dependent lytic Epstein-Barr virus reactivation in oral keratinocyte cells (60). X-box binding protein 1 (XBP-1) is suggested to play a role in the reactivation of KSHV during B-cell differentiation to plasma cells (61, 62). Similarly, knockdown of c-Myc, which likely affects the cell differentiation program, has been shown to trigger KSHV reactivation (63). These key transcriptional factors associated with cell differentiation frequently regulate the landscape of histone modification to reprogram cellular gene expression through recruitment of histone-modifying enzyme complexes. As ZIC2 is an important transcription factor for neuronal cell differentiation and cell differentiation is known to trigger herpesvirus reactivation (60, 64–66), it would be interesting to see how ZIC2 expression is regulated and explore its connection between cell differentiation and the KSHV life cycle.

In summary, we identified ZIC2 to be a critical regulator of KSHV latency maintenance. Degradation of ZIC2 by K-Rta favors lytic gene expression and triggers KSHV reactivation. Our work presents a regulatory loop in the latent-lytic switch and strengthens the importance of maintaining bivalent chromatin on the latent KSHV genome. Identification of ZIC2 cofactors or other cellular regulators should help us better understand the regulation of the KSHV life cycle.

## MATERIALS AND METHODS

### Cell culture.

BCBL-1, BC3 (ATCC), DG-75 (ATCC), and Ramos (ATCC) cells were grown in RPMI 1640 medium supplemented with 15% fetal calf serum (FBS). TREx-F3H3-K-Rta BCBL-1 cells were cultured in RPMI 1640 medium containing 15% FBS, 10 μg/ml blasticidin, and 200 μg/ml hygromycin B. iSLK.219 cells were maintained in Dulbecco modified Eagle medium (DMEM) supplemented with 10% FBS, 10 μg/ml puromycin, 1200 μg/ml hygromycin B, and 800 μg/ml G418. We are grateful to Jae Jung (University of Southern California) for kindly providing the TREx-F3H3-K-Rta BCBL-1 cell line and to Don Ganem (Novartis Institutes for Biomedical Research) for the BCBL-1 and iSLK.219 cell lines. 293 (ATCC) and 293T (ATCC) cells were grown in DMEM containing 10% FBS.

### Plasmids.

Plasmids expressing the K-Rta wild type and mutants were described previously (20). For construction of pcDNA3.1-ZIC2, a codon-optimized and shRNA-resistant ZIC2 cDNA was synthesized (Integrated DNA Technologies) and cloned into pcDNA3.1 with FLAG or HA epitope tags. For construction of the ZIC2-expressing lentivirus vector, the same cDNA was cloned into the pLenti-C-Myc-DDK vector (Origene) at the MluI and SgfI sites. For protein expression in insect cells, the cDNAs of interest were cloned into the pFastBac vector (Invitrogen).

### Generation of TREx-K-Rta His-ubiquitin 293 cells.

The inducible pcDNA5 FRT/TO FLAG×3-HA×3-K-Rta expression construct was first generated by cloning of 3×FLAG and 3×HA tandem epitope tags fused to the N-terminal region of K-Rta. Inducible 293 cells were subsequently generated by cotransfection of pcDNA5 FRT/TO FLAG×3-HA×3-K-Rta and pOG44 (Flp recombinase) into Flp-In TREx-293 cells following the manufacturer's protocol (Invitrogen). The K-Rta-inducible 293 cells were then used to create cells stably expressing His-ubiquitin by transfection of pcDNA-His-ubiquitin. Stable clones were selected in the presence of zeocin for 3 weeks. Several clones were picked, and His-ubiquitin expression in the presence or absence of MG132 (10 μg/ml) was confirmed. Multiple clones were then pooled and used for these studies.

### Identification of His-ubiquitin-conjugated proteins.

TREx-K-Rta His-ubiquitin 293 cells were cultured in DMEM containing 10% FBS. K-Rta expression was induced by addition of doxycycline at a final concentration of 1 μg/ml for 24 h. Cells grown in 10 150-mm dishes were harvested and lysed in denaturing lysis buffer (6 M guanidinium-HCl, 10 mM Tris-HCl, pH 8.0, 100 mM sodium phosphate buffer, pH 8.0, 5 mM β-mercaptoethanol, 10 mM imidazole). Nickel affinity purification of His-ubiquitin conjugates was performed with Ni-NTA agarose beads (Qiagen) according to a published protocol (67) with minor modifications. Briefly, Ni-NTA agarose beads were added to the cell lysate and mixed at 4°C for 16 h. The beads were sequentially washed with lysis buffer, pH 8.0, wash buffer (8 M urea, 10 mM Tris-HCl, pH 8.0, 100 mM sodium phosphate buffer, pH 8.0, 5 mM β-mercaptoethanol, 5 mM imidazole), and pH 6.3 wash buffer (8 M urea, 10 mM Tris-HCl, pH 8.0, 100 mM sodium phosphate buffer, pH 6.3, 5 mM β-mercaptoethanol, 5 mM imidazole). Proteins were eluted in elution buffer (8 M urea, 10 mM Tris-HCl, pH 8.0, 100 mM sodium phosphate buffer, pH 8.0, 5 mM β-mercaptoethanol, 200 mM imidazole). Proteins were digested with trypsin, desalted with StageTips, and subjected to LC-MS/MS analysis. Tandem mass spectra were compared with the spectra in the UniProt human reference proteome database using X! Tandem (version Sledgehammer; The GPM). The Scaffold program (version 4.4.3; Proteome Software) was used to validate MS/MS-based peptide and protein identifications.

### Lentivirus production and transduction.

For ZIC2-knockdown experiments, the following shRNA sequences from Sigma pLKO.1 shRNA libraries were used: GCAACTGAGCAATCCCAAGAA (shZIC2_1) and GCCGAGATGCAGGACCGTGAA (shZIC2_2).

Lentiviruses from the ZIC2-expressing lentivirus vector, ZIC2-targeting shRNAs, and nontargeting scrambled shRNA (catalog number 1864; Addgene) were produced in 293T cells. The vectors were cotransfected with psPAX2 (catalog number 12260; Addgene) and pMD2.G (catalog number 12259; Addgene) using the Lipofectamine 2000 reagent. The supernatants were collected at 48 and 72 h posttransfection. Viral particles were filtered, concentrated, and stored at −80°C until use. Cells were infected with lentiviruses in the presence of 8 μg/ml Polybrene with occasional shaking. Cells were incubated for 72 h to allow protein expression or knockdown. A control lentivirus vector expressing green fluorescent protein (GFP) was used to monitor the transfection and transduction efficiency.

### IP and Western blotting.

Cells were lysed in immunoprecipitation (IP) lysis buffer (25 mM Tris-HCl, pH 7.4, 150 mM NaCl, 1% NP-40, 1 mM EDTA, 5% glycerol) containing protease inhibitors (Roche). Total cell lysates (500 μg) were incubated with antibodies (2 μg) overnight at 4°C. Protein A/G agarose beads were used to capture antibody-protein complexes at 4°C for 3 h. The beads were then washed five times with IP lysis buffer and boiled in SDS-PAGE loading buffer for Western blotting. The antibodies used for IP and Western blotting are listed in Table 1.

**TABLE 1:** Antibodies used in this study

Antibody	Supplier	Catalog no.	Application^*a*^
ZIC2	Abcam	Ab150404	WB, ChIP-Seq, ChIP, IP
HA	Sigma	H6908	WB, IP
FLAG	Sigma	F3165	WB
His	Millipore	05-949	WB
ORF45	Santa Cruz	sc-53883	WB
K8.1	Santa Cruz	sc-65446	WB
H3	Abcam	Ab1791	ChIP
AcH3	Millipore	06-599	ChIP
H3K4me3	Millipore	07-473	ChIP
H3K27me3	Millipore	07-449	ChIP
EZH2	Cell signaling	5246s	WB, ChIP, IP
SUZ12	Abcam	Ab12073	WB, ChIP, IP
EED	Millipore	09-774	WB
Actin	Sigma	A1978	WB
IgG	Santa Cruz	sc-2027	ChIP, IP
K-Rta	Izumiya Lab		WB, IFA
K-bZIP	Izumiya Lab		WB

a WB, Western blotting; ChIP-Seq, chromatin immunoprecipitation coupled with massively parallel sequencing; ChIP, chromatin immunoprecipitation; IP, immunoprecipitation; IFA, immunofluorescence assay.

### Purification of recombinant protein and *in vitro* interaction assay.

FLAG- or GST-tagged K-Rta and FLAG-tagged ZIC2 proteins were purified from recombinant baculovirus-infected Sf9 cells according to the procedure described previously (68). For purification of PRC2 complex proteins, recombinant baculoviruses expressing FLAG-EZH2, FLAG-EED, or FLAG-SUZ12 were prepared with a Bac-to-Bac system (Invitrogen), and Sf9 cells were coinfected with all three recombinant baculoviruses. Recombinant proteins were purified from a 100-ml culture of infected Sf9 cells with anti-FLAG agarose beads (Sigma). For *in vitro* interaction assays, purified FLAG-ZIC2 and purified FLAG-PRC2 complex were incubated in binding buffer (20 mM HEPES, pH 7.9, 150 mM NaCl, 1 mM EDTA, 4 mM MgCl_2_, 0.02% NP-40, 10% glycerol, and 1 mg/ml bovine serum albumin [BSA] supplemented with 1 mM phenylmethylsulfonyl fluoride) for 90 min at 4°C. The mixture was incubated with antibodies (2 μg) overnight at 4°C and then with protein A/G agarose beads at 4°C for 3 h. The beads were washed five times with binding buffer, and the complexes were released by heat denaturation in SDS-PAGE loading buffer for analysis by Western blotting. For *in vitro* GST pulldown assays, GST or GST-tagged K-Rta protein bound to glutathione-Sepharose beads was incubated in binding buffer with purified FLAG-ZIC2 protein for 90 min at 4°C. The beads were washed five times with binding buffer, and the complexes released by heat denaturation in SDS-PAGE loading buffer were subjected to Western blotting.

### IFAs.

Immunofluorescence assays (IFAs) were performed as described previously (38). Cells on a coverslip were incubated with anti-K-Rta rabbit antibody (1:1,000 dilution) in blocking buffer (1% BSA, 0.1% Tween 20 in phosphate-buffered saline [PBS]) for 1 h at room temperature. After washing four times with PBS, the cells were incubated with Alexa Fluor 647-conjugated anti-rabbit immunoglobulin antibody (Invitrogen) in blocking buffer for 1 h at room temperature. The coverslips were washed four times with PBS, stained with Hoechst 33342 for 5 min, and mounted with SlowFade Gold antifade reagent (Invitrogen). Cells were imaged by using a Keyence BZ-X710 fluorescence microscope.

### ChIP.

Chromatin immunoprecipitation (ChIP) assays were performed as described previously (69). Briefly, cells were fixed in 1% formaldehyde for 10 min at room temperature and quenched with glycine. DNA was sonicated to an average size of ∼300 bp using a Diagenode Bioruptor. After precleaning with BSA-blocked protein A/G Dynabeads (Invitrogen), chromatin was incubated with antibodies at 4°C overnight. The chromatin immunocomplexes were collected with BSA-blocked Dynabeads. Immunoprecipitated chromatin was eluted from the beads by heating for 30 min at 65°C in elution buffer (50 mM Tris-HCl, pH 8.0, 10 mm EDTA, 1% SDS). DNA was reverse cross-linked at 65°C overnight and purified with a PCR purification kit (Qiagen). The antibodies used in the ChIP assays are listed in Table 1.

### ChIP-Seq analysis.

Briefly, chromatin DNA from 1 × 10^8^ cells was used for each immunoprecipitation assay with 10 μg of ZIC2 antibody (catalog number ab150404; Abcam). ChIP-enriched and input DNA samples (1 ng) were used to generate Illumina-compatible libraries with a Kapa LTP library preparation kit (catalog number KR0453; Kapa Biosystems) according to the manufacturer's recommendations. Libraries were submitted for sequencing (50-bp, single read) on an Illumina HiSeq 3000 sequencing system. The ChIP-Seq data (FASTQ sequence reads) were aligned to the human hg19 reference genome sequence and the reference KSHV genome sequence (human herpesvirus 8 strain JSC-1 clone BAC16 [GenBank accession number GQ994935.1]) with the Bowtie (version 2) program (70). Peak finding was performed with the MACS2 program (71) utilizing the parameters and commands described in the developer's manual. We used the default settings with a minimum FDR (*q*-value) cutoff value of 0.05. The peaks and read alignments were visualized using the Integrative Genomics Viewer (IGV) browser from The Broad Institute (72). The genomic distribution of ZIC2-binding sites was analyzed by peak annotation and visualization (PAVIS) (73). Both the upstream and the downstream lengths were set to 3 kb flanking the gene transcript start and termination sites. *De novo* motif discovery was performed on sequences ±50 bp from the centers of the ZIC2-binding peaks using the MEME suite of programs (74). Peak overlap was analyzed by use of the BEDTools tool set (75).

### Quantification of KSHV virion DNA.

KSHV virion DNA was prepared using a QIAamp MinElute virus spin kit as described previously (69). Quantification was performed by RT-qPCR using an ORF73 expression plasmid to determine the absolute copy number.

### RT-qPCR.

Total RNA was prepared with an RNeasy Plus minikit (Qiagen). cDNAs and DNAs were measured by qPCR using SYBR green PCR master mix (Thermo Fisher Scientific). The primers used for the KSHV transcripts were described previously (76). The level of gene expression was normalized to that of the cellular GAPDH signal. The primers used for ZIC2 gene expression and the ChIP assays are listed in Table 2.

**TABLE 2:** List of primers

Primer application and gene or region	Sequence	
	Forward	Reverse
RT-qPCR primers		
ZIC2	GTCCACACCTCCGATAAGCC	CTCATGGACCTTCATGTGCT
GAPDH	TCGCTCTCTGCTCCTCCTGTTC	CGCCCAATACGACCAAATCC
ChIP-qPCR primers		
Peak 1/ORF8	ATCAACACCACGCTAAACGC	TGGGCTGCCAGACCAAATA
Peak 2/ORF9	ATAACCCTGTCATCCAGTCCG	CCAGCACGAAGCGCCTAAT
Peak 3	TACCAGCAGTGAACCGACCAG	CGCCCTTCAGTGAGACTTCGT
Peak 4	TTTCTTCCTCGACAGGTCTTC	ATATCACTGCGGCCCAACT
Peak 5/K6	TGTGGGCTTTGAGTTCTGTC	GGGTCGTGGGAATATGGTAC
Peak 6	CAGGAGACGGCATTTGACC	TTCCCGAGTTGACCCAGTA
Peak 7	CACTGCCGCATTATTGTCTG	GCTCCATCGCCTCTTTCGT
Peak 8	CCTAACCCACGTCACATCAC	CAGGTAACAAAGCCGCAGA
Peak 9/ORF45	GTGCGTTTATGAGCGGAGTT	ACTTGGCTGGGATTGGTTC
Peak 10	CCGCTAACGAGGTACAGGA	GACGCCCTTGCGAACACTT
Peak 11	CGTAACCCAGCAGTGACCT	TTGGATAGACGGCTTGGAG
Peak 12/K9	CAGCGGTGTCTGTGGGATT	GGAAGGCTGGTGGTTTGGT
Peak 13	CAGGAAACATAGAAGTTGCGGA	TCTTGTCACTCTGTCCGGGTA
Peak 14/K13	GTTGCGTTGGTTGGGAGTT	CGATCTGAGCCATTGAAGC
Peak 15/LANA	ATAAGTCAGCCGGACCAAGC	GGGCACCAATCAGAAAGTAGC
ORF22	CAGCAGTGGTGTTCCCTCT	GTCGCCTTCTGTGGCTCTA
ORF64	GACCCGCTACGACCATTCT	GAACTGTGATCCCGCCTCTT
ORF50 promoter_A	TGTTTCCACGGTAATGTCG	TTTGCGGTCCACTCTATCC
ORF50 promoter_B	GGCGACGTATGCGTGAGAT	CACCGAACCAGGCAACACA
ORF50 promoter_C	TATTTCCCACGACATACTTCC	TCCGACGATGAGTATGATTGA
ORF50 promoter_D	GCAAGCAAGCTGGTGTTCT	TTGGATACCCTGGTGATGC
ORF50 promoter_E	GCATGACTCCAACCGTCTC	GGCGGGAGCTTAGCTTGAT
ORF50 promoter_F	CTGACCCACCAAGGAGAAC	GTGCCACCAATGTATGACC
ORF50 promoter_G	AGCCAGCGTATGCTTCAGG	CCACATCGGGTTTCGTCAA
ORF50 promoter_H	GCTACCGGCGACTCATTAA	TGCCTGGACAGTATTCTCACA
ORF50 promoter_I	CGGGTGTCAGGGCTCTTAT	CTGAAAAGCACCGCCCGAT

### *In vitro* ubiquitin conjugation.

*In vitro* ubiquitination assays were performed as described previously (20). Ubiquitin E1 (UBE1), ubiquitin E2 (UbcH5a), and HA-ubiquitin were obtained from a commercial source (Boston Biochem). FLAG-ZIC2 was used as a substrate. Ubiquitin conjugation reactions were performed using a final protein concentration of E1 of 60 nM, E2 of 300 nM, E3 (K-Rta) of 100 nM, and ubiquitin of 10 μM in ubiquitin conjugation reaction buffer supplemented with an energy regeneration solution (Boston Biochem) for 2.5 h at 37°C.

### Statistical analysis.

Results are shown as means ± standard deviations (SDs) from at least three independent experiments. Data were analyzed using unpaired Student's *t* test. A *P* value of <0.05 was considered statistically significant.

### Accession number(s).

The sequencing data for the ChIP-Seq study can be found in the NCBI Gene Expression Omnibus database under accession number GSE102462.

## Supplementary Material

Supplemental material
