# Supplementary material for: ZIC2 Is Essential for Maintenance of Latency and Is a Target of an Immediate Early Protein during Kaposi's Sarcoma-Associated Herpesvirus Lytic Reactivation
Source: J Virol. 2017 Oct 13;91(21):e00980-17. doi: 10.1128/JVI.00980-17 (PMC5640855; doi:10.1128/JVI.00980-17)
Supplement: Supplemental material [file supp_91_21_e00980-17__index.html]

Supplemental material 

# ZIC2 Is Essential for Maintenance of Latency and Is a Target of an Immediate Early Protein during Kaposi's Sarcoma-Associated Herpesvirus Lytic Reactivation

## Supplemental material

- Supplemental file 1 -

  Table S1 (Proteins with a spectral count of >10 and a >1.4-fold change between samples from untreated and Dox-treated TREx-KRta His-Ubiquitin 293 cells.)

  PDF, 89K
