# Supplementary material for: ZIC2 Is Essential for Maintenance of Latency and Is a Target of an Immediate Early Protein during Kaposi's Sarcoma-Associated Herpesvirus Lytic Reactivation
Source: J Virol. 2017 Oct 13;91(21):e00980-17. doi: 10.1128/JVI.00980-17 (PMC5640855; doi:10.1128/JVI.00980-17)
Supplement: Supplemental material [file JVI.00980-17_zjv999183038s1.pdf]

Table S1. Proteins with spectral count >10 and fold change >1.4 between samples from untreated and Dox-treated TREx-K-Rta His-Ubiquitin 293 cells.

#	Protein name	Spectral count	
		No Dox	+ Dox
1	Transcriptional repressor protein YY1 OS=Homo sapiens GN=YY1 PE=1 SV=2	1105	755
2	Zinc finger CCCH domain-containing protein 18 OS=Homo sapiens GN=ZC3H18 PE=1 SV=2	662	428
3	60S ribosomal protein L27a OS=Homo sapiens GN=RPL27A PE=1 SV=2	329	203
4	RNA-binding protein with serine-rich domain 1 (Fragment) OS=Homo sapiens GN=RNPS1 PE=1 SV=1	256	178
5	CLK4-associating serine/arginine-rich protein OS=Homo sapiens GN=CLASRP PE=1 SV=1	185	96
6	Serine/arginine-rich splicing factor 6 OS=Homo sapiens GN=SRSF6 PE=1 SV=2	135	194
7	Histone H2A type 2-C OS=Homo sapiens GN=HIST2H2AC PE=1 SV=4	134	92
8	Bcl-2-associated transcription factor 1 OS=Homo sapiens GN=BCLAF1 PE=1 SV=1	132	200
9	Histone H2B type 1-K OS=Homo sapiens GN=HIST1H2BK PE=1 SV=3	111	75
10	Transcription factor GATA-6 OS=Homo sapiens GN=GATA6 PE=1 SV=2	92	57
11	Protein FAM76B OS=Homo sapiens GN=FAM76B PE=1 SV=1	88	60
12	Nucleolar and coiled-body phosphoprotein 1 OS=Homo sapiens GN=NOLC1 PE=1 SV=2	77	113
13	Histone-binding protein RBBP7 OS=Homo sapiens GN=RBBP7 PE=1 SV=1	71	125
14	Nucleolar and coiled-body phosphoprotein 1 OS=Homo sapiens GN=NOLC1 PE=1 SV=2	66	163
15	E3 SUMO-protein ligase CBX4 OS=Homo sapiens GN=CBX4 PE=1 SV=3	64	40
16	Chromodomain-helicase-DNA-binding protein 8 OS=Homo sapiens GN=CHD8 PE=1 SV=5	62	36
17	Zinc finger protein ZIC 2 OS=Homo sapiens GN=ZIC2 PE=1 SV=2	61	26
18	Pinin OS=Homo sapiens GN=PNN PE=1 SV=4	56	19
19	Zinc finger protein ZIC 5 OS=Homo sapiens GN=ZIC5 PE=1 SV=2	55	33
20	Zinc finger protein 281 OS=Homo sapiens GN=ZNF281 PE=1 SV=1	51	21
21	RNA-binding protein 33 OS=Homo sapiens GN=RBM33 PE=1 SV=3	50	21
22	Zinc transporter ZIP10 OS=Homo sapiens GN=SLC39A10 PE=1 SV=2	49	31
23	Nucleolin OS=Homo sapiens GN=NCL PE=1 SV=3	47	76

24	Calcium homeostasis endoplasmic reticulum protein OS=Homo sapiens GN=CHERP PE=1 SV=3	44	63
25	Thyroid hormone receptor-associated protein 3 OS=Homo sapiens GN=THRAP3 PE=1 SV=2	42	69
26	Nucleolin OS=Homo sapiens GN=NCL PE=1 SV=3	40	5
27	p21-activated protein kinase-interacting protein 1 OS=Homo sapiens GN=PAK1IP1 PE=1 SV=2	40	23
28	Zinc finger protein 485 OS=Homo sapiens GN=ZNF485 PE=2 SV=1	39	25
29	Zinc finger and SCAN domain-containing protein 26 OS=Homo sapiens GN=ZSCAN26 PE=1 SV=1	38	26
30	40S ribosomal protein S7 OS=Homo sapiens GN=RPS7 PE=1 SV=1	35	22
31	Zinc transporter ZIP6 OS=Homo sapiens GN=SLC39A6 PE=1 SV=3	32	13
32	Sentrin-specific protease 1 OS=Homo sapiens GN=SENP1 PE=1 SV=2	29	19
33	Leucine-rich repeat and calponin homology domain-containing protein 1 OS=Homo sapiens GN=LRCH1 PE=1 SV=3	28	16
34	CLK4-associating serine/arginine rich protein OS=Homo sapiens GN=CLASRP PE=1 SV=4	28	50
35	RNA/RNP complex-1-interacting phosphatase OS=Homo sapiens GN=DUSP11 PE=1 SV=1	27	17
36	Hepatoma-derived growth factor-related protein 2 OS=Homo sapiens GN=HDGFRP2 PE=1 SV=1	26	40
37	Zinc finger protein 239 OS=Homo sapiens GN=ZNF239 PE=2 SV=3	26	15
38	E3 ubiquitin-protein ligase Arkadia OS=Homo sapiens GN=RNF111 PE=1 SV=3	25	13
39	Transcription factor AP-2-alpha OS=Homo sapiens GN=TFAP2A PE=1 SV=1	25	17
40	Actin, alpha cardiac muscle 1 OS=Homo sapiens GN=ACTC1 PE=1 SV=1	24	16
41	Zinc finger protein 655 OS=Homo sapiens GN=ZNF655 PE=1 SV=3	24	15
42	NADH dehydrogenase [ubiquinone] 1 beta subcomplex subunit 3 OS=Homo sapiens GN=NDUFB3 PE=1 SV=3	24	14
43	Zinc finger protein 649 OS=Homo sapiens GN=ZNF649 PE=1 SV=1	24	14
44	Putative RNA-binding protein Luc7-like 2 OS=Homo sapiens GN=LUC7L2 PE=1 SV=2	22	11
45	Dual specificity tyrosine-phosphorylation-regulated kinase 1A OS=Homo sapiens GN=DYRK1A PE=1 SV=2	21	14
46	Exportin-2 OS=Homo sapiens GN=CSE1L PE=1 SV=3	20	47
47	Glyceraldehyde-3-phosphate dehydrogenase OS=Homo sapiens GN=GAPDH PE=1 SV=1	20	7
48	Zinc finger protein 417 OS=Homo sapiens GN=ZNF417 PE=2 SV=2	20	14
49	Splicing factor, proline- and glutamine-rich OS=Homo sapiens GN=SFPQ PE=1 SV=2	18	3
50	Heat shock protein beta-1 OS=Homo sapiens GN=HSPB1 PE=1 SV=2	18	5
51	Homeobox protein Hox-A9 OS=Homo sapiens GN=HOXA9 PE=1 SV=4	16	4
52	Prickle-like protein 3 OS=Homo sapiens GN=PRICKLE3 PE=1 SV=2	15	8
53	Keratin, type II cytoskeletal 1 OS=Homo sapiens GN=KRT1 PE=1 SV=6	15	10

54	Transducin-like enhancer protein 3 OS=Homo sapiens GN=TLE3 PE=1 SV=1	15	30
55	Superoxide dismutase [Mn], mitochondrial OS=Homo sapiens GN=SOD2 PE=1 SV=2	15	31
56	Nucleoside diphosphate-linked moiety X motif 19, mitochondrial OS=Homo sapiens GN=NUDT19 PE=1 SV=1	15	7
57	Homeobox protein DLX-2 OS=Homo sapiens GN=DLX2 PE=1 SV=2	15	8
58	Leucine-rich repeat-containing protein 59 OS=Homo sapiens GN=LRRC59 PE=1 SV=1	14	4
59	Keratin, type I cytoskeletal 9 OS=Homo sapiens GN=KRT9 PE=1 SV=3	14	6
60	Transcription factor MafB OS=Homo sapiens GN=MAFB PE=1 SV=2	14	4
61	Plasminogen activator inhibitor 1 RNA-binding protein OS=Homo sapiens GN=SERBP1 PE=1 SV=2	14	22
62	Homeobox protein SIX1 OS=Homo sapiens GN=SIX1 PE=1 SV=1	14	9
63	DNA-dependent protein kinase catalytic subunit OS=Homo sapiens GN=PRKDC PE=1 SV=3	13	24
64	Calnexin OS=Homo sapiens GN=CANX PE=1 SV=2	13	19
65	Keratin, type II cytoskeletal 2 epidermal OS=Homo sapiens GN=KRT2 PE=1 SV=2	12	3
66	Serine/arginine-rich splicing factor 3 OS=Homo sapiens GN=SRSF3 PE=1 SV=1	11	5
67	Arginine-glutamic acid dipeptide repeats protein OS=Homo sapiens GN=RERE PE=1 SV=2	11	4
68	Peroxisome proliferator-activated receptor gamma coactivator-related protein 1 OS=Homo sapiens GN=PPRC1 PE=1 SV=1	11	1
69	Alpha-S1-casein OS=Bos taurus GN=CSN1S1 PE=1 SV=2	11	3
70	Serine/threonine-protein kinase PRP4 homolog OS=Homo sapiens GN=PRPF4B PE=1 SV=3	10	16
71	Pre-mRNA-splicing factor CWC22 homolog OS=Homo sapiens GN=CWC22 PE=1 SV=3	9	13
72	Stromal interaction molecule 1 OS=Homo sapiens GN=STIM1 PE=1 SV=3	9	18
73	Protein transport protein Sec16A OS=Homo sapiens GN=SEC16A PE=1 SV=1	9	13
74	Queuine tRNA-ribosyltransferase subunit QTRTD1 OS=Homo sapiens GN=QTRTD1 PE=1 SV=1	9	14
75	G patch domain-containing protein 8 OS=Homo sapiens GN=GPATCH8 PE=1 SV=2	8	16
76	Lamina-associated polypeptide 2, isoforms beta/gamma OS=Homo sapiens GN=TMPO PE=1 SV=2	8	17
77	Zinc phosphodiesterase ELAC protein 2 (Fragment) OS=Homo sapiens GN=ELAC2 PE=1 SV=2	6	11
78	Cyclic AMP-responsive element-binding protein 5 OS=Homo sapiens GN=CREB5 PE=1 SV=3	2	15
79	Titin OS=Homo sapiens GN=TTN PE=1 SV=1	1	13
80	Serine/arginine repetitive matrix protein 2 (Fragment) OS=Homo sapiens GN=SRRM2 PE=1 SV=1	0	18
81	DNA topoisomerase I, mitochondrial OS=Homo sapiens GN=TOP1MT PE=1 SV=1	0	20
82	Methyl-CpG-binding protein 2 OS=Homo sapiens GN=MECP2 PE=1 SV=1	0	15
